# Transient neonatal pustular melanosis: An unusual and challenging eruption

**DOI:** 10.1002/ccr3.8092

**Published:** 2023-10-25

**Authors:** Michelle Marie Boffa, Janine Borg, Marie‐Claire Grech, David Pace, Simon Attard Montalto

**Affiliations:** ^1^ Mater Dei Hospital Msida Malta

**Keywords:** bullae, dermatology, neonatal dermatosis, neonatology, pediatrics

## Abstract

TNPM is a benign, transient, neonatal pustulosis requiring no active treatment. Diagnosis is clinical, characterized by a vesiculopustular eruption, healing with residual hyperpigmented macules. Before diagnosing TNPM, serious conditions including skin infections should be excluded. This case was unusual in that vesiculobullae predominated, with a notable absence of simple pustules.

## Graphical abstract

Flacid bullae and desquamation over the scalp and back of a 1 hour old neonate.

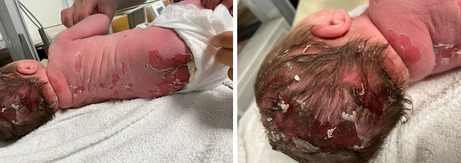

## 1 CASE PRESENTATION

**FIGURE 1:**
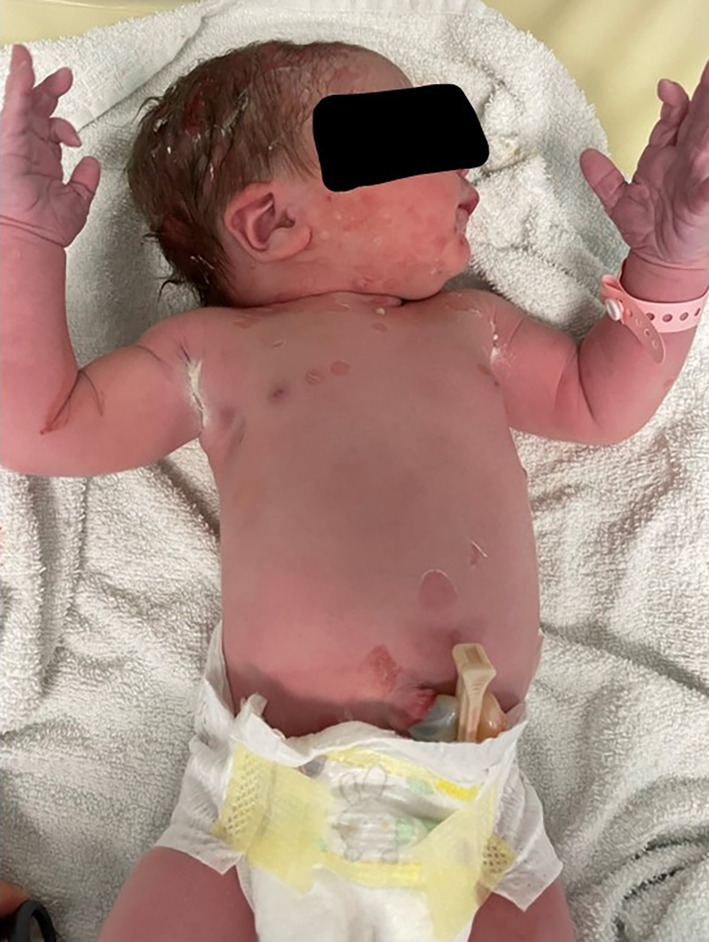
Image taken at 1 hour of life depicting areas of desquamation with a mixture of vesicles and bullae that were either flaccid, ruptured or purulent, over the trunk, face, head, and neck.

**FIGURE 2:**
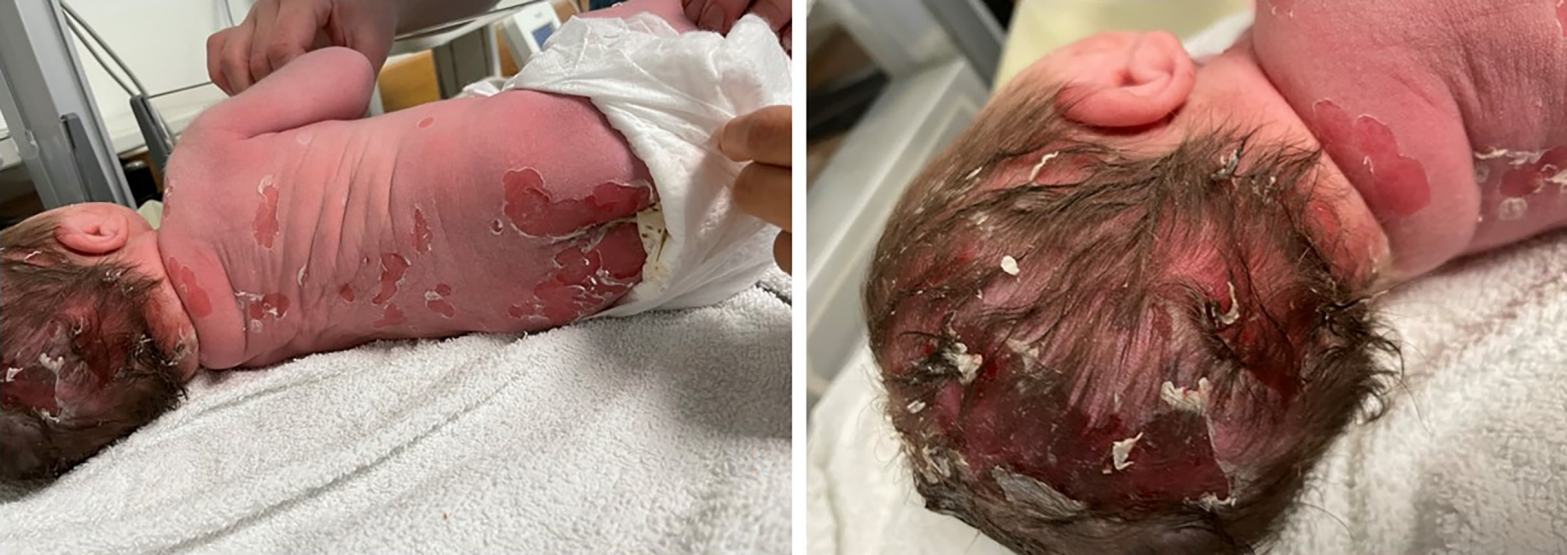
Images of the scalp and back taken at 1 hour of life showing flaccid bullae, including one 8 mm in diameter in the right scapular area and patchy desquamation over the back and annular desquamation over the scalp.

**FIGURE 3:**
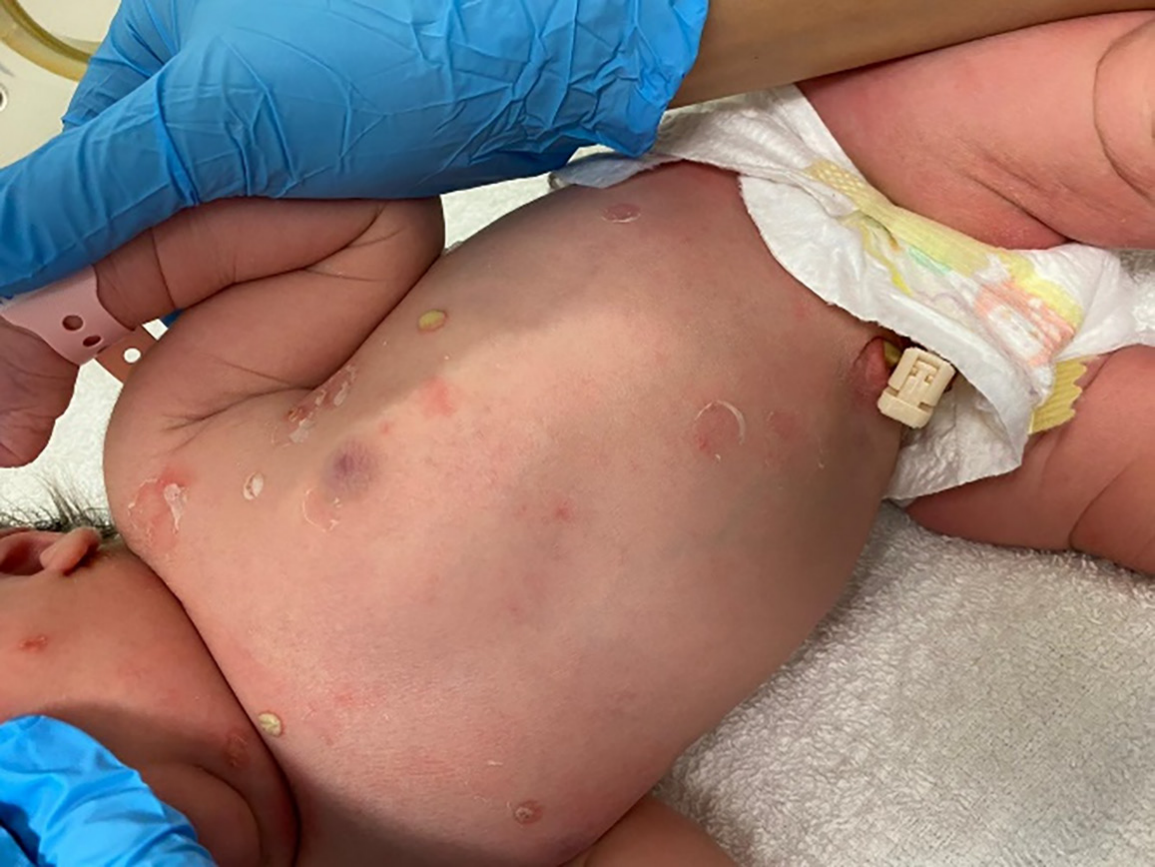
Image taken at a few days of life showing sparse bullae and vesicles, with erythematous patches at previous sites of bullae.

A term, female neonate, with no maternal risk factors for sepsis, was born via an uncomplicated, noninstrumental, vaginal delivery following an uneventful pregnancy. There was no family history of blistering disorders and no history of consanguinity. At birth, she was noted to have a rash that consisted of generalized desquamation over the face, head, neck, trunk, and limbs (including the digits but excluding the palms and soles) with vesicles and bullae, ranging from 3 to 8 mm in diameter, some flaccid and some containing white fluid. There was also evidence of ruptured bullae with residual erythematous, raw skin, but no small, simple pustules (Figures 1 and 2). Of note were patches of desquamation over the back, with a large patch over the buttocks, and some annular desquamated lesions over the scalp (Figure 3). The mucosae and conjunctivae were unaffected.

She was otherwise systemically well, with no abnormalities on examination. She was self‐ventilating in air, with no need for respiratory support, and she remained hemodynamically stable. The feed volume was optimized early on in view of the risk of transcutaneous fluid losses and subsequent dehydration.

The initial diagnosis included a neonatal blistering disorder or staphylococcal/streptococcal skin infection and, for this reason, she was transferred to the neonatal intensive care unit and started on broad‐spectrum intravenous antibiotics (cefotaxime and amoxicillin). Initial bloods were unremarkable, blood cultures were negative, and a swab of the fluid in one of the vesicles was sterile. At Day 5 of life, following input from the pediatric infectious diseases and dermatology teams, a diagnosis of transient neonatal pustular melanosis (TNPM) was suggested and a bullous aspirate sent for cytology and varicella zoster PCR. The latter was negative, but cytology demonstrated an abundance of neutrophils, eosinophils and occasional macrophages and keratinocytes, with no viral cytopathic changes or bacterial or fungal elements. Antibiotics were discontinued after 7 days and, having remained well, she was discharged on day seven of life, with advice to apply emollients.

On follow‐up, she remained well with no further lesions developing beyond 4 weeks of age. On review at age 6 weeks, there was residual discoloration at the site of some of the lesions, but complete resolution was documented at the next visit a few months later, further confirming the clinical diagnosis of TNPM.

## 2 DISCUSSION

### 2.1 What is TNPM?

TNPM is a benign neonatal skin condition presenting at birth. The etiology is still unknown, but there is no familial predisposition or association with maternal infection or drug exposure.^1^, ^2^ It is uncommon, with an incidence ranging from 0.6% in white infants to 4.4% in black infants, with no sex predilection.^3^ TNPM is self‐limiting and usually characterized by a generalized eruption of sterile pustules,^3^ although in our case the eruption was primarily bullous.

Diagnosis is predominantly clinical, with typical features including a generalized pustular eruption in an otherwise healthy term neonate with no systemic malaise.^1^, ^2^, ^3^ Lesions usually measure 1 mm–5 mm in diameter and have a predilection for the forehead, temporal areas, cheeks, neck, back, and buttocks.^3^ The palms and soles are often spared.^3^ An important diagnostic feature is that the skin in between the lesions is visually healthy and non‐erythematous.^1^, ^3^

### 2.2 Natural history

The natural history is one of pustules and vesicles initially appearing during the first hours or days of life. These then rupture to develop residual pigmented macules with collarette scaling.^3^, ^4^, ^5^ In fact, the co‐existence of pustules and pigmented macules, on a background of unaffected skin, is typical of TNPM. In our patient, there were no obvious pustules, but the eruption was mostly vesiculobullous, with some containing sterile fluid and existing as both intact and ruptured lesions, sparing the palms and soles. TNPM is transient, requiring no specific treatment. Initial skin lesions usually disappear spontaneously within the first 2 weeks, with the residual hyperpigmented scaly macules persisting for several weeks to months.^2^, ^3^, ^4^ In some, neonates TNPM may present at birth with hyperpigmented macules, with no pustules or vesicles, due to an in utero vesicular stage.^5^

### 2.3 Diagnosis and differential diagnosis

Although the diagnosis is clinical, in cases of diagnostic uncertainty, screening for other pathologies requiring urgent medical attention is indicated.^1^, ^3^ Investigations include a peripheral blood count, blood film and culture, pustular fluid gram staining, microscopy, and culture and viral PCR tests. In TNPM, these investigations should all be normal and not yield any organisms after 48 h.^1^ Cytological examination of pustular fluid would show abundant polynuclear neutrophils and occasional eosinophils, in contrast to erythema toxicum neonatorum where infiltrates are predominantly eosinophilic.^2^, ^3^, ^6^ A skin biopsy is not usually necessary; however, if performed, this would demonstrate subcorneal or intraepidermal pustules or vesicles.^1^, ^3^

Neonatal vesiculopustular eruptions could also be due to other serious pathologies, including active bacterial infections (e.g., *Staphylococcus aureus, Streptococcus, Pseudomonas aeruginosa*, and *Listeria monocytogenes*), congenital syphilis, viral infections (herpes, varicella, and cytomegalovirus), and congenital candida infection.^2^, ^3^ Other benign, transient neonatal pustular dermatoses include erythema toxicum neonatorum, miliary pustulosis, acne neonatorum, infantile acropustulosis, and eosinophilic pustulosis.^2^, ^3^, ^4^

In some cases, such as ours, impressive clinical presentations might result in diagnostic uncertainty, leading to over‐investigation and treatment. Therefore, confident diagnosis is important to provide reassurance about TNPM's natural history. Offering follow‐up until lesion resolution would serve to monitor for the development of complications such as secondary bacterial infections, alleviate parental anxiety, and minimize irrational use of unnecessary treatment.^1^, ^3^, ^4^

## 3 CONCLUSION

TNPM is a benign neonatal dermatosis. Although the diagnosis was initially unclear due to the severity and vesiculobullous predominance of the eruption, this neonate remained systemically well throughout, without any red flags to raise any suspicion of more serious pathology. This case highlights the importance of early diagnosis of TNPM, and the avoidance of unnecessary over‐investigation and overtreatment, while providing due parental reassurance.

## AUTHOR CONTRIBUTIONS

**Michelle Marie Boffa:** Conceptualization; writing – original draft; writing – review and editing. **Janine Borg:** Writing – original draft. **Marie‐Claire Grech:** Writing – original draft. **David Pace:** Conceptualization; investigation; writing – review and editing. **Simon Attard Montalto:** Conceptualization; investigation; writing – review and editing.

## FUNDING INFORMATION

None.

## CONFLICT OF INTEREST STATEMENT

None to declare.

## CONSNET

We confirm that, to our knowledge, the information we are presenting is true and that patient confidentiality has been maintained throughout. Written informed consent was obtained from the patient to publish this report in accordance with the journal's patient consent policy.

## Data Availability

The data that support the findings of this study are available from the corresponding author upon reasonable request.Also: Written informed consent was obtained from the patient to publish this report in accordance with the journal's patient consent policy and has been uploaded as a separate file.
